# Supplementary material for: Interpretable (not just posthoc-explainable) medical claims modeling for discharge placement to reduce preventable all-cause readmissions or death
Source: PLoS One. 2024 May 9;19(5):e0302871. doi: 10.1371/journal.pone.0302871 (PMC11081343; doi:10.1371/journal.pone.0302871)
Supplement: S1 File — (PDF) [file pone.0302871.s001.pdf]

Supplement to: Interpretable (not just posthoc-explainable) medical claims modeling for discharge placement to reduce preventable all-cause readmissions or death

Ted L. Chang[1, 2], Hongjing Xia[1, 2], Sonya Mahajan[1, 2], Rohit Mahajan[1, 2], Joe Maisog[3], Shashaank Vattikuti[4], Carson Chow[5, 2], Josh Chang[2, 1, 6, *],

1 Sound Prediction Inc., Columbus OH, USA
2 Mederrata Research Inc., Columbus OH, USA
3 Lee Health, Fort Meyers FL, USA
4 Sleep Research Center, Walter Reed Army Institute of Research, Silver Spring MD, USA
5 Laboratory of Biological Modeling, NIDDK, National Institutes of Health, Bethesda MD, USA
6 Epidemiology and Biostatistics Section, Rehabilitation Medicine Department, The National Institutes of Health, Besthesda MD, USA

* josh.chang@nih.gov

Supplementary Methods

Medicare data preprocessing

Here we describe some details on the choices we made in preprocessing that will help make our work reproducible. Kyle Barron's Medicare Documentation repository of Medicare data documenation is an excellent resource for acquainting oneself with this standardized dataset. Our first steps in processing the CMS LDS were to merge the files, originally organized by year, into long tables for each claim type. In the process, we renamed pre-2011 columns in the dataset to match 2011+ plus columns where-ever they differed. We will refer to the dataset using 2011 and beyond column names.

Episode Grouping

The CMS LDS consists of records organized into claims. Multiple claims can constitute a single period or episode of service. We determined episodes of the following types:

1. inpatient (inp)
2. skilled nursing facility (snf)
3. hospice (hosp)
4. outpatient (out, car)

For determining episodes, we grouped claims of each of the given types by person, and sorted by either the admission date (for inp, snf, hosp), or the claim through-date for (out, car).

Then for inp, snf, hosp, we merged successive claims into running episodes if they overlapped temporally, if the provider was the same and the intermediate discharge code indicates that the individual was not otherwise discharged home in between (we allow for distinct episodes with zero days of wait if a patient is discharged home and returns on the same day).

For out and car, we did the same merging with all claim types together, relaxing the need for the provider to match in an episode. Then we filtered for out/car episodes that did not overlap with inp, snf, hosp episodes – we determined these to be true outpatient episodes.

Then, for out and inp episodes, we determined if they corresponded to emergency department visits by looking for corresponding revenue center codes.

Model Specification

Parameter	Decomposition	Max order
$\boldsymbol{\alpha}$	MDC × Hx × CC/MCC	2
$\boldsymbol{\beta}$	race	1
$\boldsymbol{\gamma}$	MDC × Hx × CC/MCC	2
$\boldsymbol{\nu}$	MDC × Hx × CC/MCC	2

Table 1. Specific decompositions used per parameter to define cohorts, where major diagnostic category (MDC) is of size 26, history (Hx) is of size 2^5, corresponding to low/high in each of the five dimensions, CC/MCC is of size 3, and race is of size 5.

The specific decompositions that we used for each of the model terms are displayed in Fig. 1. For the missing parameter $\boldsymbol{\xi}$, the results in this manuscript are all determined using $\boldsymbol{\xi} = \mathbf{0}$.

The python package `bayesianquilts`, with demonstration available at `github:mederrata/bayesianquilts` contains utilities for managing decompositions such as these.

We used a regularized horseshoe prior in order to encourage $\boldsymbol{\beta}$ to be sparse. Specifically, we applied an independent horseshoe prior to this parameter within every model cohort.

The individual components of each of the parameter decompositions were all modeled using Gaussian weakly-informative priors (with a default scale of 5 for the zero-order terms in the expansion). We helped encourage shrinkage by having the scale of these priors decay for higher order terms in the decomposition. For the results in the paper, we used a decay factor of 0.1 per each order.

Training

We utilize TFP's ADVI routines, which utilize stochastic sampling in computation of the ELBO. For this reason, it is not uncommon for specific parameter combinations to be in highly improbable locations – which can trigger underflows. To avoid instabilities, re adjust the likelihood on a per-observation level, first computing the minimum finite value of the log likelihood and then setting any divergent values to the minimum finite value minus a fixed offset of 100. We use the soft-plus function as a default bijector for any parameters that are supposed to be non-negative.

SHAP

KernelSHAP, the general all-purpose model-agnostic implementation of SHAP, is very resource intensive, so we had to tune it in order to run it. First, we used the regression-based version of KernelSHAP, found in the python package `shapreg` [**?**], which is not as resource intensive.

Second, although we had a very powerful computational resource at our disposal (Pittsburgh Supercomputer Center Bridges 2-AI with 512GB RAM), we had to restrict

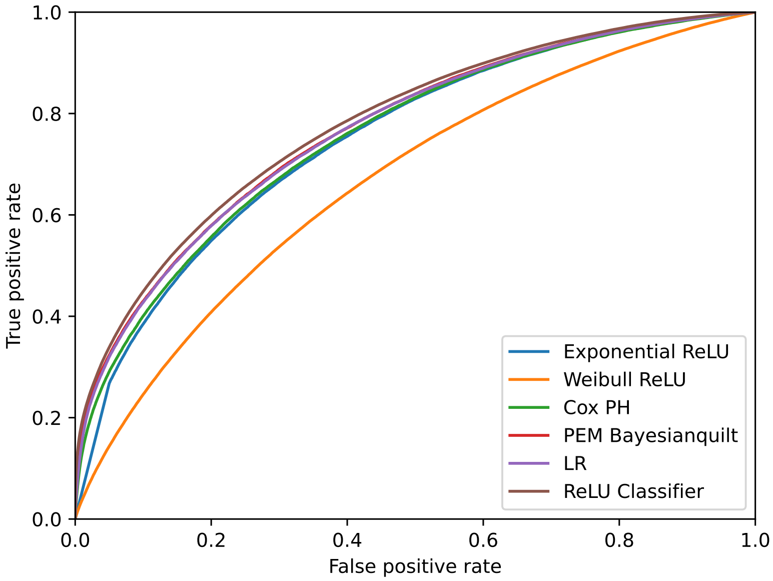

Fig S1. ROC Curves for select models. Note that the curves of the best-performing models substantially overlap and are difficult to distinguish.

the input data size to 5k random training examples. Otherwise, we found that the system would run out of memory, causing the application to segmentation fault.

Finally, in order to get around an error involving a singular matrix, we regularized the linear algebra problem embedded within the algorithm, adding a fixed small constant of 10^{-8} to the diagonal of the linear transformation matrix (see) for the exact modification made.

We were able to run `shapref` with `n_samples=2400` and `batch_size=24` on our resource in approximately 5 hours. Due to the memory requirement issues with our large dataset, we are not able to scale this result to more data. SHAP is known to be computationally expensive, particularly for large datasets and a large number of features (see github issues 1053 1495), and its very computation is inherently based on approximations. We believe that our computation of SHAP for our model is a reasonable representation of how well-approximated it is in practice, on a real problem and on a real dataset with a large number of predictors.

Our main point in the main text is a reiteration of the well-known fact that SHAP feature importance is not guaranteed to match what a model is doing in practice when the features in the training data are correlated – as would be true in most real-world problems. Examining Fig. 5 in the context of Fig. S7, one sees that the most-important SHAP features are not themselves very important in the model.

Supplementary Results

Here are results omitted from the main text for space constraints.

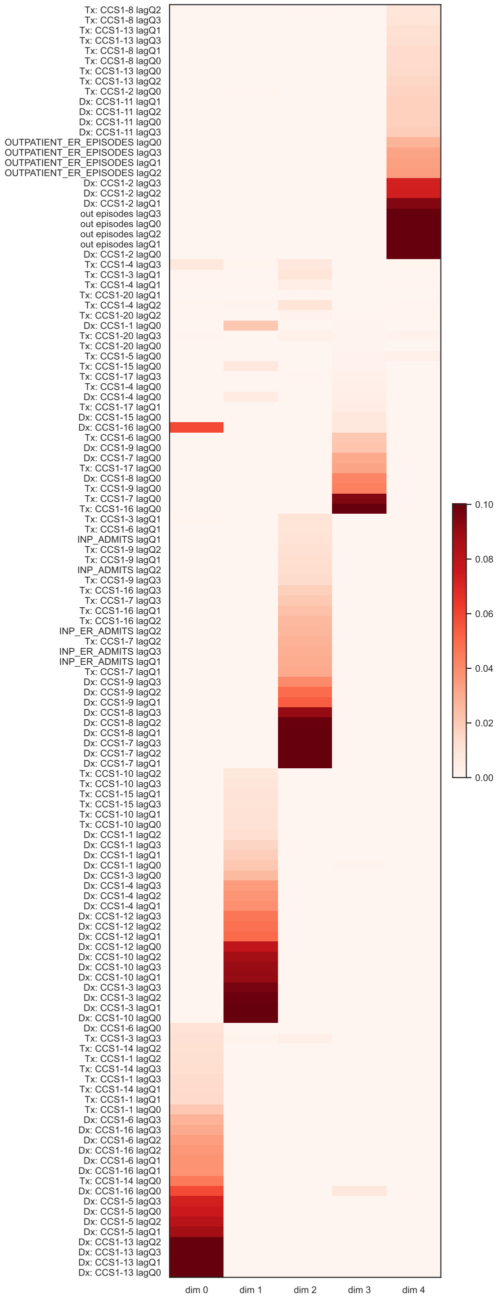

Fig S2. Historical quarterly utilization count encoding with up to 25 features per dimension

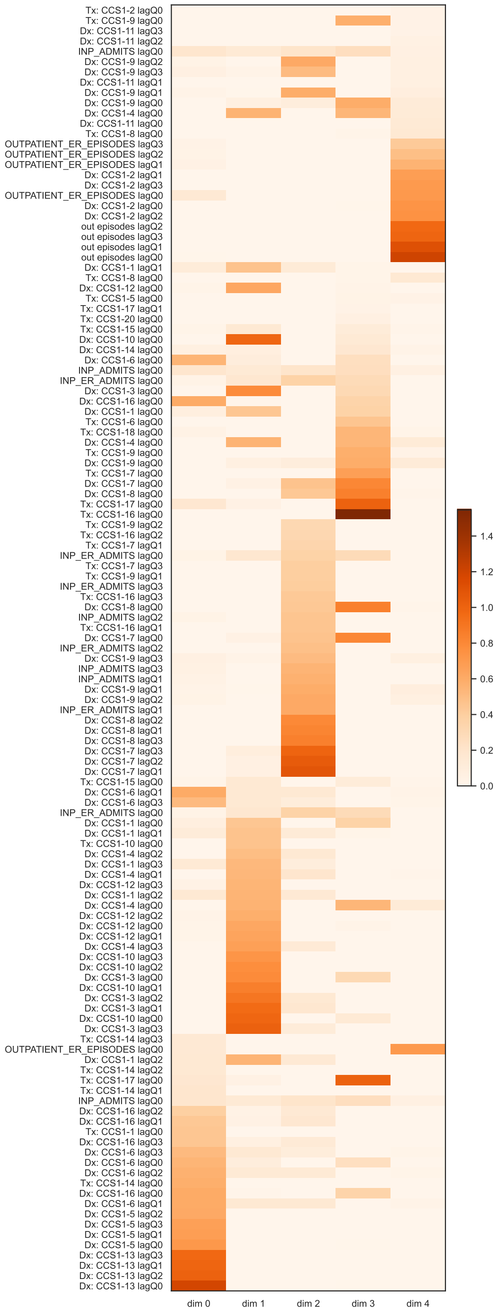

Fig S3. Decoding matrix corresponding to the encoding model of Fig. S2 showing up 25 features per dimension

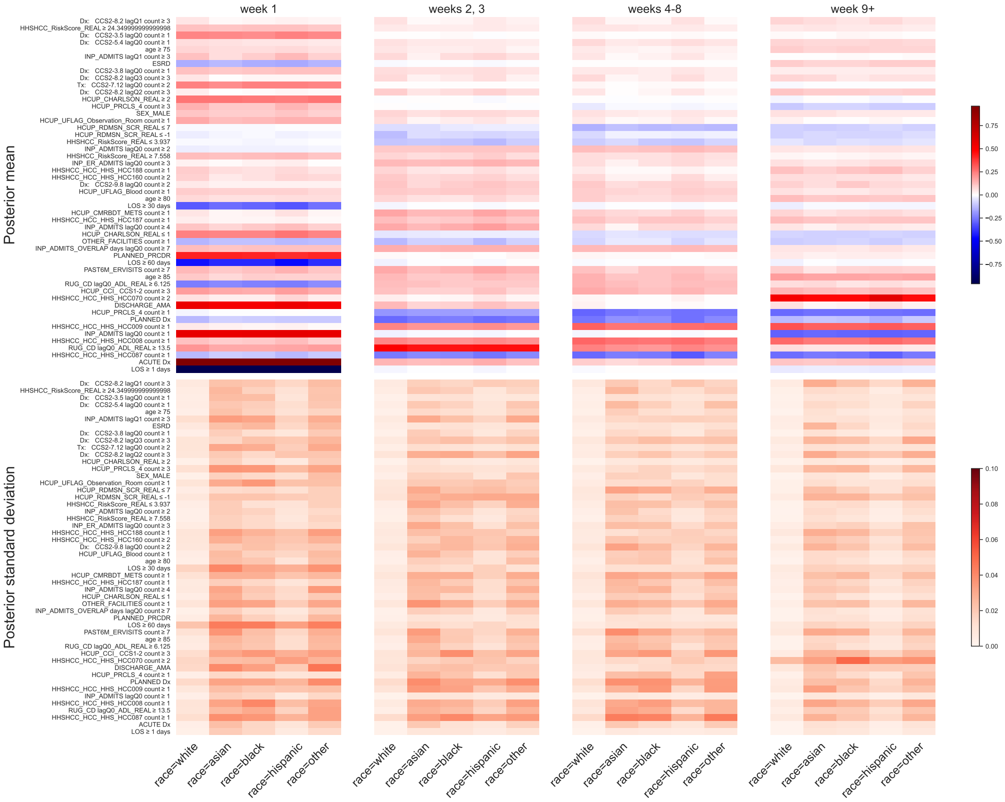

Fig S4. The 50 most influential regressors $\boldsymbol{\beta}$ (posterior mean, standard deviation) tracked through all time intervals. A more-comprehensive version of this figure can be found in our other supplemental file.

History representation

We utilized sparse probabilistic matrix factorization in order to obtain a low-dimension representation of personal medical history for the year prior to each episode. The encodings given by the model specify linear combinations of the original data features that define a representation of an episode's history. The representations then can be constituted into a predictive distribution for the original features by transformation against a decoding matrix (Fig. S3). Note that this method finds a subset of the input features that can be used to predict the value of all features.

Random slopes

Although we do not use this terminology in the main text, in the language of hierarchical mixed effects models the parameters $\boldsymbol{\beta}$, $\boldsymbol{\xi}$ in the model are random slopes. In the main text we presented the week 1 slopes in Fig. 3 In Fig. 4, we present the components of $\boldsymbol{\beta}$ of the largest magnitudes, across all time intervals. As we noted in the main text, length of stay being at least 1 day, or conversely, being less than a full day, was the most impact predictor of early readmission. However, the effect disappears after one week. Long length of stay (greater than 30 days) appeared to follow the same trend, with those having a length of stay of at least a month having a lower readmission risk in the first week after discharge, but not reduced risk after the first week. Generally, the magnitude of the slopes tended to increase over time, with a few exceptions.

Random intercepts

The parameter $\boldsymbol{\alpha}$ from Eq. 4 is specific to each cohort in the model – it is a random intercept in hierarchical mixed effects modeling terminology. We presented the posterior mean for this parameter in Fig. 2 in the main text, interpreting this quantity as a cohort-specific baseline survival.

Causal inference

In our model we adjust for treatment selection bias by incorporating estimates of the treatment probabilities as covariates. The ordinal logistic regression intercepts are provided in Fig. S5.

The first five components in the parameter $\boldsymbol{\gamma}$ adjust for the selection bias present in claims. We present our cohort-specific estimates of $\boldsymbol{\gamma}$ in Fig. S6. We present the full timecourse of discharge placement effects in Fig. S7. Largely, the discharge placement affects appear to strengthen from week 1 to weeks 2/3 before weakening from week four onwards. The discharge placement bias effects have more cohort-level variability after the first week. In Fig. S8, we zoom in on the effects for the cohorts that benefit the most from the discharge placement interventions. A key advantage of this form of modeling against even the computationally interpretable ReLU-nets is the ability to perform mesoscopic cohort-level inference and interpretation. Cohort-level information facilitates making a model actionable since actions can be applied to subgroups all at once.

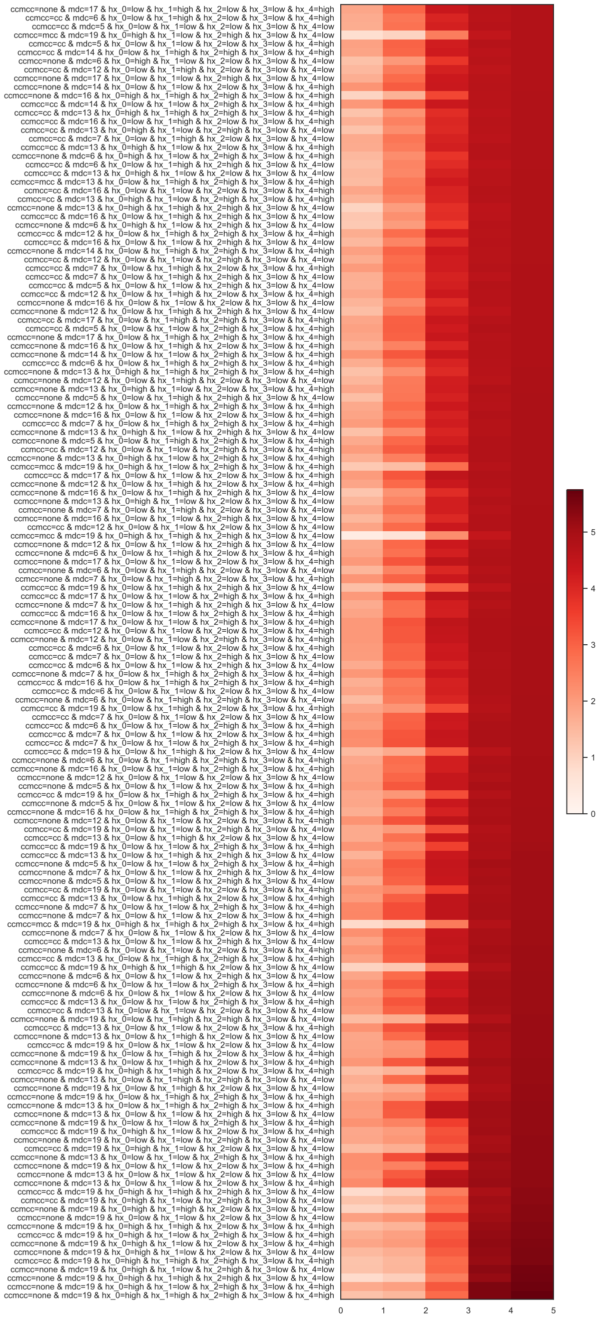

Fig S5. Cohort-wise ordinal intercept terms ν for the prediction of the distribution of discharge assignments.

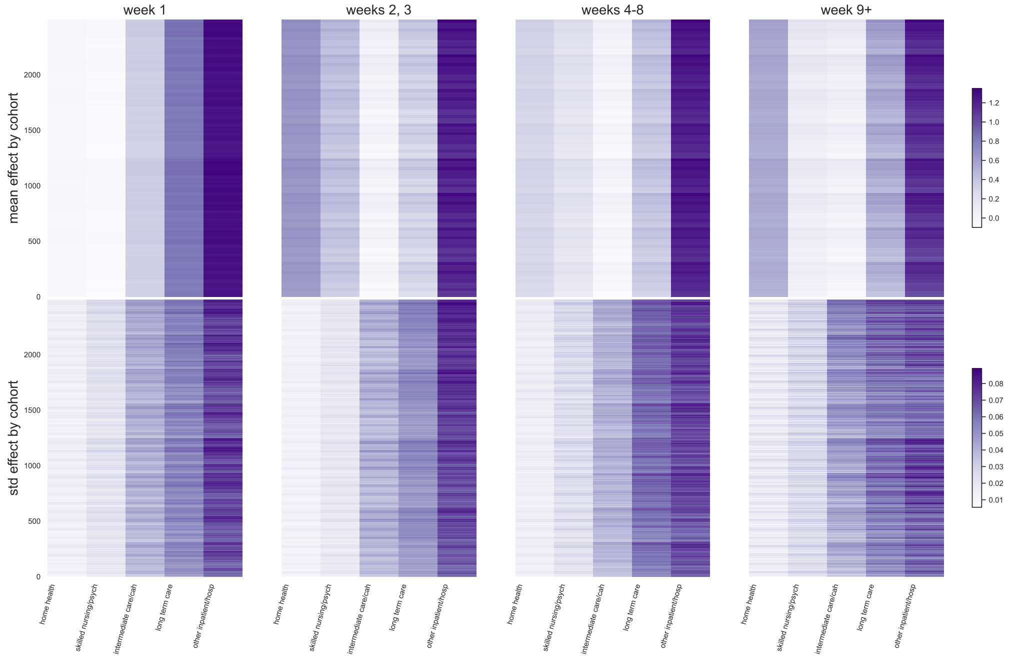

Fig S6. Discharge assignment adjustment terms corresponding to first five terms of γ (posterior mean, standard deviation) in terms of log hazard ratio under the log-additive effects model of Eq. **??**

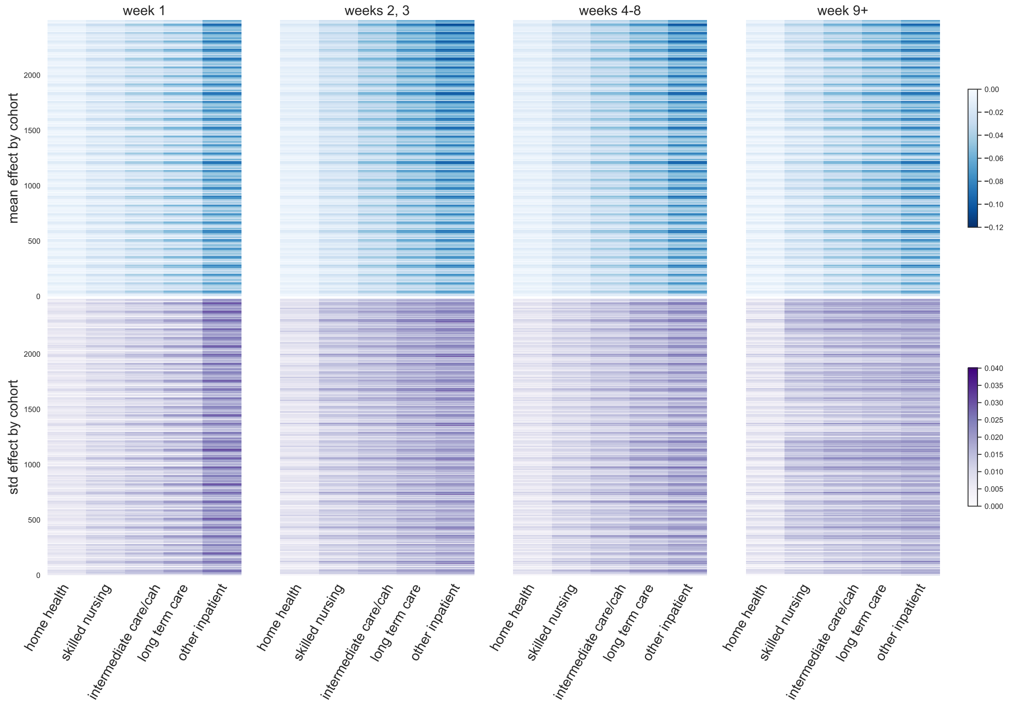

Fig S7. Discharge placement effects γ (posterior mean, standard deviation) in terms of log hazard ratio under the log-additive effects model of Eq. **??**

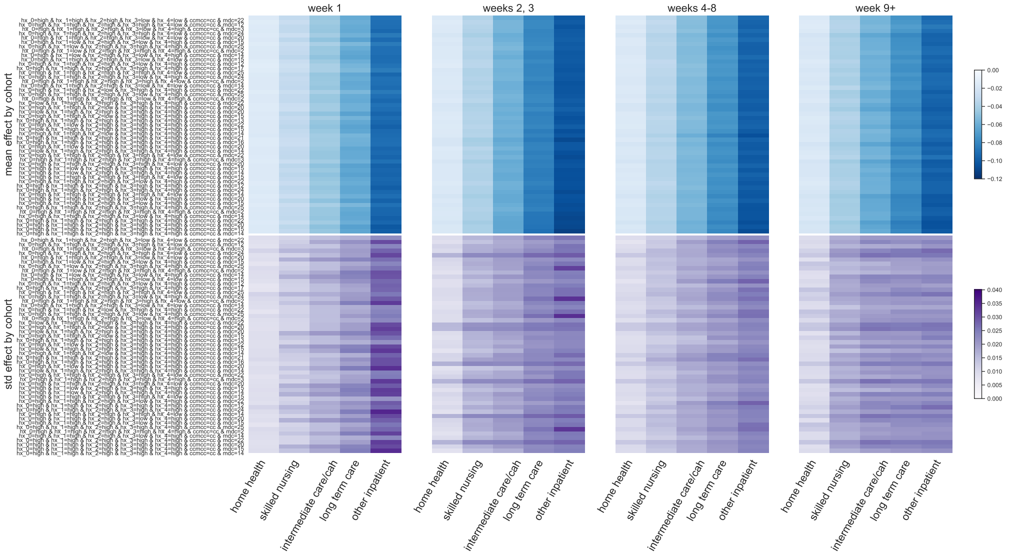

Fig S8. Discharge placement effects for select cohorts with the largest mean discharge placement effects.

Please see Supplement S2 file for review history for this manuscript at KDD.
