# Supplementary material for: Interpretable (not just posthoc-explainable) medical claims modeling for discharge placement to reduce preventable all-cause readmissions or death
Source: PLoS One. 2024 May 9;19(5):e0302871. doi: 10.1371/journal.pone.0302871 (PMC11081343; doi:10.1371/journal.pone.0302871)
Supplement: Attachment — Submitted filename: review_reply.pdf [file pone.0302871.s003.pdf]

We thank the reviewers and editor for spending the time to evaluate our work.

Here is a summary of our main changes:

- We updated the citations in the reference list - many were previously preprints that are now published
- We incorporated many additional recent citations on the use of xAI in healthcare, particularly for readmission
- We made the changes the editor requested

Review #1

3. The Discussion does not compare and contrast with the existing literature. It would be helpful for the reader to comprehensively understand how the investigators' model compares to existing models on a more detailed level.

Thanks to reviewer 1 for this suggestion. We added the following text to place our modeling methodology in context of the literature:

Critical look at the applied literature

As evidenced by the explosion of applied machine learning manuscripts that claim explainability or interpretability, the research community has realized that it is important to have some understanding of what a machine learning model is doing. In many cases, the claim of interpretability is warranted, for instance in manuscripts that use methods such as logistic regression or even more-advanced methods such as explainable-boosting machines [70, 71]. However, a large volume of studies (a small sampling for example [72, 73, 74, 75, 76]) are in-actuality putting forward blackboxes as explainable by using SHAP or similar methodologies. Additionally, many manuscripts eschew the word explainable altogether and claim that their SHAP-endowed blackboxes are interpretable. It is our humble opinion that the machine learning field needs to set a higher bar for what should be labeled explainable, let along interpretable. It is our hope that we have made a contribution to this particular conversation.

4. The Limitations section should be expanded and more specific.

We have added the following text:

Our modeling approach has downsides. Numerical stability when performing ADVI inference generally requires the use of double precision floating point – a limitation common to Bayesian inference (popular statistical packages such as Stan use double precision by default). This limitation is significant when looking to expand to larger models that encompass more data features. The methodology is based on piecewise linear modeling defined over a high-dimensional lattice of coarsened variables derived from the data. For this reason, models can become big quickly and can run into practical memory constraints, particularly in unison with the requirement for using double precision floating point. The trend in machine learning has

been to move towards more automation and the search for modeling architectures that do not require tuning beyond engineering of input predictors. Bucking this trend, our framework is designed with more intentionality in mind – it requires the practitioner to think about what types of broad interactions make sense for a given problem and what types of coarsening will give rise to a model that is understandable and useful in a real-world sense. Some may view this characteristic as a limitation.

Reviewer #2:
- Since the AUC ROC is the main measure of performance. I was hoping to see the AUCROC curvers for running the proposed model and the other compared models for multiple running points (TPRs, and FDRs).

We originally omitted the curves since it is graphically difficult to distinguish between the curves of the best-forming models (the piecewise models in addition to the neural net classifier and XG-boost). We now include curves for some select models in the Supplemental Materials. For your convenience, we replicated the figure here:

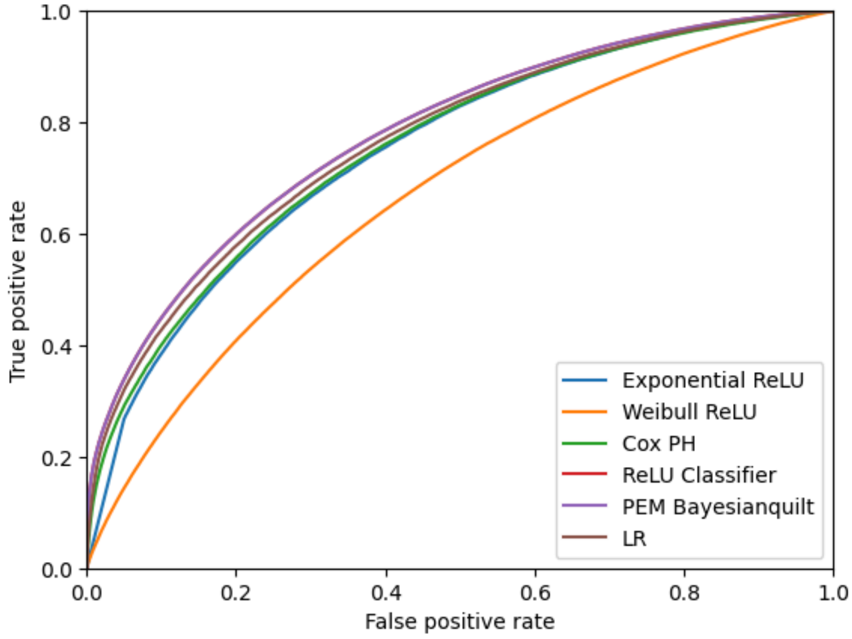

- The abstract may add quantity of the samples (around 1.2 million episodes) with/instead of the 5% of the data.

Done

- For the scope of health care, I suggest to highlight more applications of XAI in health care. I suggest to highlight PMID: 38066735 and/or PMID: 38132885

We have taken the opportunity to update our XAI citations, including those mentioned by the reviewer.

- Please order the references [1],[2], ascendingly based on the appearance in the manuscript.

Done
