# Supplementary material for: Interpretable (not just posthoc-explainable) medical claims modeling for discharge placement to reduce preventable all-cause readmissions or death
Source: PLoS One. 2024 May 9;19(5):e0302871. doi: 10.1371/journal.pone.0302871 (PMC11081343; doi:10.1371/journal.pone.0302871)
Supplement: S2 File — (PDF) [file pone.0302871.s002.pdf]

← Back to **the profile of Joshua C Chang** (/profile?id=~Joshua_C_Chang1)

Interpretable (not just posthoc-explainable) medical claims modeling for discharge placement to reduce preventable all-cause readmissions or death (/pdf?id=SA6vy0ozPUI)

Joshua C Chang (/profile?id=~Joshua_C_Chang1), Ted Chang (/profile?email=ted%40mederrata.com), Carson Chow (/profile?email=carson.chow%40nih.gov), Rohit Mahajan (/profile?email=ro%40mederrata.com), Sonya Mahajan (/profile?email=sonya%40mederrata.com), Joe Maisog (/profile?email=bravas02%40gmail.com), Shashaank Vattikuti (/profile?email=shashaank76%40gmail.com), Hongjing Xia (/profile?email=hongjing%40mederrata.com)

28 Jan 2023 (modified: 16 May 2023) Submitted to KDD 2023 - ADS Readers: Applied Data Science Track, Paper82 Area Chairs, Paper82 Reviewers, Paper82 Authors Show Bibtex Show Revisions (/revisions?id=SA6vy0ozPUI)

Keywords: medical claims, readmission, survival, bayesian, nonparametric, discharge, interpretability, explainability

TL;DR: We demonstrate an interpretable readmission model that performs as well as blackbox models and use it to give an example of how posthoc-xAI misleads

Abstract: We developed an inherently interpretable multilevel Bayesian framework for representing variation in regression coefficients that mimics the piecewise linearity of ReLU-activated deep neural networks. We used the framework to formulate a survival model for using medical claims to predict hospital readmission and death that focuses on discharge placement, adjusting for confounding in estimating causal local average treatment effects. We trained the model on a 5% sample of Medicare beneficiaries from 2008 and 2011, based on their 2009--2011 inpatient episodes, and then tested the model on 2012 episodes. The model scored an AUROC of approximately 0.76 on predicting all-cause readmissions -- defined using official Centers for Medicare and Medicaid Services (CMS) methodology -- or death within 30-days of discharge, being competitive against XGBoost and a Bayesian deep neural network, demonstrating that one need-not sacrifice interpretability for accuracy. Crucially, as a regression model, we provide what blackboxes cannot -- the exact gold-standard global interpretation of the model, identifying relative risk factors and quantifying the effect of discharge placement. We also show that the posthoc explainer SHAP fails to provide accurate explanations.

Submission Guidelines: Yes

Student Author: No

Serve As Reviewer: Joshua Chang josh@mederrata.com

Add **Withdraw**

Reply Type: all Author: everybody Visible To: all readers **10 Replies**

Hidden From: nobody

[−] **Paper Decision**

KDD 2023 Conference Applied Data Science Track Program Chairs

14 May 2023 KDD 2023 Conference Applied Data Science Track Paper82 Decision Readers: Program Chairs, Paper82 Authors Show Revisions (/revisions?id=MaWn9Q1_MLA)

Decision: Reject

Comment: Thank you for submitting an interesting paper to KDD

Unfortunately the paper would not directly be useful in reducing readmissions in its current form (I will briefly describe my concerns*); the paper does not provide new clinically relevant knowledge that was mined from the data that could be useful to the clinical community; the methodology is limited and not compared against sufficient ML baselines to understand the real impact of best in class black box (non explainable) methods; it does not really compare against widely used (albeit simplistic) 30 day readmission risk scores like LACE and HOSPITAL models; and does not demonstrate how it would be used in process improvements that can drive reduction of readmissions/death (there are many such national process improvement efforts that are well known in the clinical community working on reducing readmissions).

NOTE*: claims data are generated for billing as opposed to clinical purposes. They are produced and validated much after the actual discharge time for the patient. They lack sufficient details (eg labs) to provide actionable intervention in real time while the patient is in the hospital pre-discharge (to improve the discharge notes for the particular patient) or to improve community care post discharge. For these reasons the analysis of such data is not primarily for the purposes of intervening on patients; thus explainability is not the primary motivation. The analysis of such data is more for statistical purposes eg to normalize and compare different populations which may have some economic or other differences. It is not at all apparent to this reviewer why explainability is even useful under these circumstances.

For all these reasons we are unable to recommend accepting this paper to the KDD program at this time.

[−] **Reply to reviewers**

KDD 2023 Conference Applied Data Science Track Paper82 Authors Joshua C Chang (/profile?id=~Joshua_C_Chang1) (privately revealed to you)

13 Apr 2023 KDD 2023 Conference Applied Data Science Track Paper82 Official Comment Readers: Paper82 Reviewers Submitted, Paper82 Authors, Program Chairs, Paper82 Area Chairs Show Revisions (/revisions?id=ErOiItkycfE)

Comment:
We thank the reviewers for the comments.

We are aware that this manuscript doesn't follow the formulae most-commonly found in machine learning papers: in particular the reviewers may have found our baselines to be lacking. However, we think that for a real-life problem one should resist evaluation of a solution based solely on bake-off type metrics. Over-optimization of left-out model metrics is not guaranteed to translate to real-world performance. However, we have compared our model to the most-common types of readmission models found in the literature (NN with one hidden layer, XGBoost), evaluating those models on the same dataset/labels. We also note that our performance metrics are good relative to that reported in the literature.

We are working to improve manuscript readability. What has made writing this manuscript challenging is that we are trying to do quite a few things 1) introduce the problem 2) differentiating being intrinsic interpretability and bandaids such as xAI 3) introducing the modeling methodology, which relates hierarchical mixed effects regression to RELU-nets, finding commonality between the two 4) describing the scientific findings which are numerous and readily available since the model, as a regression model, is interpretable 5) providing a SHAP counterexample.

In the updated manuscript we have removed Fig 2 and Fig 3, shortening the text and giving us space for clarifying the rationale behind our modeling choices and on how cohorts within the dataset are defined.

[−] **Official Review of Paper82 by Reviewer HX1W**

KDD 2023 Conference Applied Data Science Track Paper82 Reviewer HX1W

24 Mar 2023 KDD 2023 Conference Applied Data Science Track Paper82 Official Review Readers: Program Chairs, Paper82 Area Chairs, Paper82 Reviewers Submitted, Paper82 Authors Show Revisions (/revisions?id=T6BWuz35_3)

Note: I have read and agree with the ADS track's policy on behalf of myself.

Paper Category: Data and Benchmarks

Influence: Yes, the authors showed statistics/results of how users have benefited from the solution.

Novelty: 4

Technical Soundness: Mostly, but with minor flaws (e.g., missing references)

Impact: A good solution to a rather narrowly defined problem for a small target group of users

Presentation: Mostly clear, with minor readability issues

Reproducibility: Yes - Good. It provides reasonably complete information that will help reproduce the results.

Summary:
This paper presents an inherently interpretable multilevel Bayesian to predict hospital readmissions and death after discharge.

Paper Strength:
1 The description is clear. 2 The methodology is sound and feasible. 3 The result interpretation is sufficient and clear.

Paper Weakness:
The paper is too long.

Detailed Evaluation And Suggestions For Authors:
The authors should make the paper shorter and concise.

Overall Evaluation: 3: An OK paper, good enough for KDD (weak accept). Accept if possible, although I would not be upset if it were rejected.

Confidence Level: 3: I have read key papers in this area.

[−] **Reply to reviewer HX1W**

KDD 2023 Conference Applied Data Science Track Paper82 Authors Joshua C Chang (/profile?id=~Joshua_C_Chang1) (privately revealed to you)

13 Apr 2023 KDD 2023 Conference Applied Data Science Track Paper82 Official Comment Readers: Program Chairs, Paper82 Area Chairs, Paper82 Reviewers Submitted, Paper82 Authors Show Revisions (/revisions?id=sYqj9NOJrr-)

Comment:
Thank you for the favorable review. We appreciate your concern about paper length. We intend this manuscript to differ from the standard machine learning manuscript in a key way: because of model interpretability, we are able to make concrete scientific findings – it is our intention to explore those findings to some extent. That said, we are making an effort to condense some portions of the manuscript as appropriate, including by moving Fig 2 to the supplement and removing Fig 3, which is available in the supplement in larger form.

This model is trained on nationwide medical claims data and gives insights that are useful to both providers and payers. For this reason we do not believe the target group of users to be small.

[−] **Official Review of Paper82 by Reviewer Am5N**

KDD 2023 Conference Applied Data Science Track Paper82 Reviewer Am5N

23 Mar 2023 (modified: 23 Mar 2023) KDD 2023 Conference Applied Data Science Track Paper82 Official Review Readers: Program Chairs, Paper82 Area Chairs, Paper82 Reviewers Submitted, Paper82 Authors Show Revisions (/revisions?id=GP2H07Ku5tF)

Note: I have read and agree with the ADS track's policy on behalf of myself.

Paper Category: Data and Benchmarks

Influence: Yes, the authors have designed their solution for a particular audience (but no statistics/results of how the audience benefit yet).

Novelty: 3

Technical Soundness: Mostly, but with minor flaws (e.g., missing references)

Impact: A good solution to a rather narrowly defined problem for a small target group of users

Presentation: Mostly clear, with minor readability issues

Reproducibility: Yes - Good. It provides reasonably complete information that will help reproduce the results.

Summary:
The manuscript aims to formulate a survival model for using medical claim data to predict hospital readmission and death. The authors aimed to developed an interpretable multilevel Bayesian framework for representing variation in regression coefficients that mimics the piecewise linearity of ReLU-activated deep neural networks. The authors claimed that their proposed method has better interpretablility but yet achieve comparable performance compared to the baseline models.

Paper Strength:
1/ I agree that, in additio to prediction accuracy, interpretability is an important prospective that worth investing significant amount research effort. This is especially true for healthcare related applicaitons. If it is possible, any inference on the causality between the risk factors and the outcomes do can help our end users to understand the model better and gain better confidence over the predictive model.

2/ The proposed method is sound

3/ It has demostrated the limited effectivness of global SHAP

4/ The manuscript is generally well written

Paper Weakness:
1/ The study focused on predicting patients hospital readmission with just claim data. While it may be easier to gain access to a large amount of claim data, but the claim data is known to be limited in granularity and clinical details about the patient. In recent years, as more and more electronic health record (EHR) data become available, it may be more meaningful and useful to predict hosptial readmisssion with EHR data instea. E.g here a recent review on readmission prediction with EHR data

https://www.bmj.com/content/369/bmj.m958.abstract (https://www.bmj.com/content/369/bmj.m958.abstract)

2/ The prediction accuracy of the proposed method is benchmarked against XGboost and BNN, where BNN was implemented with a single hidden layer. I suggest the authors should conduct more extensive experiments to compare against more sophiscated "blockbox" models. This can help to convince the readers that "the proposed model is as good as the complex blockbox models but offers better interpretability"

3/ The proposed method was only evaluated over one dataset and also only over one problem statement. How well it may generalised to other datasets and other healthcare predictive problems is unclear.

Detailed Evaluation And Suggestions For Authors:
Refer to the above section

Overall Evaluation: 3: An OK paper, good enough for KDD (weak accept). Accept if possible, although I would not be upset if it were rejected.

Confidence Level: 3: I have read key papers in this area.

[−] **Reply to reviewer Am5N**

KDD 2023 Conference Applied Data Science Track Paper82 Authors Joshua C Chang (/profile?id=~Joshua_C_Chang1) (privately revealed to you)

13 Apr 2023 KDD 2023 Conference Applied Data Science Track Paper82 Official Comment Readers: Program Chairs, Paper82 Area Chairs, Paper82 Reviewers

Submitted, Paper82 Authors Show Revisions (/revisions?id=Z0idlCGZAt)

Comment:

Thank you for the very detailed review. We appreciate the point about electronic health records-based prediction. We have updated the text, adding some comments about alternatively using EHR data (including some references). When comparing EHR to claims there are tradeoffs. EHR data has more-detailed information than available in claims. The major limitation of medical claims is the lack of test results which provide a detailed picture of the decisions that a provider makes regarding discharge and treatments.

However, EHR-based models are going to be more-customized to the institutions where the records are obtained. Raw medical records are more-subject to variability in documentation practices. Additionally, the scope and breadth of the patient population reflected in a set of EHR data will not be as comprehensive as that found in claims - particularly when comparing against Medicare claims which sample every institution in the United States. On the individual basis- you also get a better picture of longitudinal information through claims than through EHR. That said, it would be best to integrate EHR and claims together, which we will be working on with a clinical partner in the coming months.

We chose to benchmark against XGBoost and deep learning because those are the most popular models found in the literature, particularly in this problem domain. Additionally, our team was one of the contestants in the inaugural CMS AI Innovations challenge though working part-time at the time we were not able to execute this new class of models in the limited time available - the finalists to that challenge submitted blackbox (XGBoost/NN/ ensemble between XGBoost+NN) - for that reason we additionally wanted to target these methodologies in this manuscript.

We are working on procuring additional datasets. We have access to a set of synthetic claims from a major US payer but are not permitted to publish on that data. We are working on access to state level all-payer databases but do not know what the timeline would be like for obtaining access.

[−]
Official Review of Paper82 by Reviewer WiAM

KDD 2023 Conference Applied Data Science Track Paper82 Reviewer WiAM

23 Mar 2023 KDD 2023 Conference Applied Data Science Track Paper82 Official Review Readers: Program Chairs, Paper82 Area Chairs, Paper82 Reviewers Submitted, Paper82 Authors Show Revisions (/revisions?id=y7D5eGf5bj)

Note: I have read and agree with the ADS track's policy on behalf of myself.

Paper Category: Data and Benchmarks

Influence: Yes, the authors showed statistics/results of how users have benefited from the solution.

Novelty: 3

Technical Soundness: No (some main elements are problematic)

Impact: Fair solution to an important problem, but there are alternatives around

Presentation: Mostly clear, with minor readability issues

Reproducibility: Yes - Good. It provides reasonably complete information that will help reproduce the results.

Summary:

This paper developed an interpretable multilevel Bayesian framework for representing variation in regression coefficients utilizing the piecewise linearity of ReLU-activated deep neural networks.

Paper Strength:

1. This paper clearly defined model interpretability under the deep learning framework.
2. For heavy-tailed problems, the authors proposed a quantile-based coding method.
3. This research is motivated by a real-world problem.

Paper Weakness:

1. For the heavy tail problem, besides quantile-based coding, are there other simple solutions that also work, such as taking the log?
2. What are the assumptions for causal inference in equation (5), for example, unctuousness?

3. How to handle the time dependence between the training dataset and test dataset?
4. This paper only applies SHAP and the proposed method to one dataset, so it is hard to make a general conclusion. Other validation sets are needed.

Detailed Evaluation And Suggestions For Authors:
Please see the comments on strengths and weaknesses.

Overall Evaluation: 2: An OK paper, but likely not good enough for KDD (weak reject). I vote for rejecting it, although I would not be upset if it were accepted.

Confidence Level: 3: I have read key papers in this area.

[–] **Reply to reviewer WiAM**

KDD 2023 Conference Applied Data Science Track Paper82 Authors Joshua C Chang (/profile?id=~Joshua_C_Chang1) (privately revealed to you)

13 Apr 2023 KDD 2023 Conference Applied Data Science Track Paper82 Official Comment Readers: Program Chairs, Paper82 Area Chairs, Paper82 Reviewers Submitted, Paper82 Authors Show Revisions (/revisions?id=HH4w-wkaUjg)

Comment:
Thanks, we are working on improving the readability of the manuscript. Addressing the enumerated weaknesses:

1. We are incorporating a discussion on the alternative transformations that we used in the updated manuscript: a) Yes, we originally tried a log-transform log(x+1) on the heavy-tailed counts, but found the performance of those models to be worse than z-score transformed models. We think this was because in many of the count variables, the most meaningful change is by going from 0 -> 1. b) c) We also tried the rank-based inverse normal z transform but decided against using it because the results are more difficult to interpret (due in-part to the heavily asymmetric nature of the distributions of the count predictors).
2. Besides citing the texts on using regression for causal inference, we have included in the manuscript some additional description of the assumptions. In Bayesian causal inference the notion of identifiability of causal effects is different from that of frequentist point estimation, however most of the same concerns hold, mainly conditional ignorability. Use of hierarchical modeling makes the positivity assumption less of a concern, and allows us the flexibility to infer heterogeneous effects. The one concern of model-based versus model-free causal inference is that our analysis depends on the model being well-specified. We believe this model is well-specified based on the benchmarks.
3. The CMS LDS sample we were given follows the same group of beneficiaries in time. If the concern is that there are people overlapping in both the train (2009-2011) and test (2012) sets, we also had the same concern. We evaluated a separate test/train split regimen early-on where we split based on person-id rather than on time – at that time we only evaluated logistic regression and XGBoost. We found the metrics to be consistent across both types of splitting so we did not further use this splitting scheme. The 2012 splitting was the type used in the evaluation of the CMS AI Health Innovations challenge so that is what we decided to compute and report on that basis.
4. We point out that SHAP is likely to be wrong re: feature attribution, anytime features are correlated. If one is evaluating the following statement, which is often taken for granted: “SHAP tells you what features a model is using,” our manuscript is a proof by contradiction - as this type of proof requires just a single counter-example. The invalidity of SHAP is not a secret - it is just under-appreciated and under-publicized.

[–] **Official Review of Paper82 by Reviewer XPbn**

KDD 2023 Conference Applied Data Science Track Paper82 Reviewer XPbn

16 Mar 2023 KDD 2023 Conference Applied Data Science Track Paper82 Official Review Readers: Program Chairs, Paper82 Area Chairs, Paper82 Reviewers Submitted, Paper82 Authors Show Revisions (/revisions?id=qfH8In35v7l)

Note: I have read and agree with the ADS track's policy on behalf of myself.

Paper Category: Scientific Discovery

Influence: Yes, the authors showed statistics/results of how users have benefited from the solution.

Novelty: 4

Technical Soundness: Mostly, but with major flaws (e.g., missing baselines)

Impact: A good solution to a rather narrowly defined problem for a small target group of users

Presentation: Hard to read, due to poor presentation

Reproducibility: Yes - Poor. Some description provided, but it is clearly insufficient information for reproducibility.

Summary:
The researchers created a naturally understandable, multi-layered Bayesian model to represent variations in regression coefficients, similar to the piecewise linearity found in ReLU-activated deep neural networks. They employed this model to develop a survival prediction method for hospital readmission and mortality, using medical claims data and concentrating on discharge placement. The approach also accounts for confounding factors in estimating local average treatment effects with a causal focus.

Paper Strength:
Considering causality is crucial when focusing on prediction. The example demonstrates that a conventional model could perform comparably to more advanced models while maintaining mechanistic interpretability. Additionally, it reveals that the SHAP criteria, a post hoc method for explainable-AI, may not always be effective in real-world scenarios.

Paper Weakness:
Rather than employing complex methods like deep learning to model the intricate relationship between the original X and Y, the authors explored transforming X to establish a simpler connection between the transformed X and Y. However, the transformation process is not explicitly detailed in the main text. For instance, the definition of "cohort" is unclear, and the reason for having five dimensions in Figure 3 is not explained. Additionally, the generalizability of the transformation to other data sets is uncertain. Some aspects of the approach, such as the number of quantiles used in feature quantization, appear to be subjective.

Detailed Evaluation And Suggestions For Authors:
The methods section needs more elaboration and clarity. Additionally, it would be beneficial to demonstrate the generalizability of the techniques while minimizing subjective decision-making.

Overall Evaluation: 3: An OK paper, good enough for KDD (weak accept). Accept if possible, although I would not be upset if it were rejected.

Confidence Level: 4: I have worked on this problem/area.

[−] **Reply to reviewer XPbn**

KDD 2023 Conference Applied Data Science Track Paper82 Authors Joshua C Chang (/profile?id=~Joshua_C_Chang1) (privately revealed to you)

13 Apr 2023 KDD 2023 Conference Applied Data Science Track Paper82 Official Comment Readers: Program Chairs, Paper82 Area Chairs, Paper82 Reviewers Submitted, Paper82 Authors Show Revisions (/revisions?id=ebNjaYVe2YY)

Comment:
Thank you for your suggestions. In the revised manuscript we are including a figure with posterior effect sizes for the most-risky cohorts plotted - where the criteria for defining the cohorts is also displayed in a friendlier manner. We note that Fig S7 provides some hints as to what constitutes a cohort but acknowledge that the definition is still unclear.

As to subjectivity, our methodology finds a way to use classic hierarchical modeling in modern big-data settings, by using the data to define scaffolding for a hierarchy. In standard mixed effects modeling, one has the coefficients vary across discrete variables in the dataset. We use the data to generate additional discrete groupings to vary coefficients, noting similarity between this method and RELU-nets, which we believe to be highly generalizable as a technique. That said, we are in many ways arguing for subjectivity in modeling. In order to obtain trustworthy results that make sense and are actionable on a human scale, human sensibilities must be baked in. This thinking made its way into some choices, for instance the choice of setting breakpoints at 1 week, 4 weeks, and 9 weeks – breakpoints that could otherwise be trained with the other variables. So in some ways we

trade some of what might be “optimal” according to a machine-based metric for what is simpler from a human perspective. Our model IS complex, but it is modular and one could fully understand it without using unreliable approximations.

About OpenReview (/about)
Hosting a Venue (/group?id=OpenReview.net/Support)
All Venues (/venues)
Sponsors (/sponsors)
Frequently Asked Questions (https://docs.openreview.net/getting-started/frequently-asked-questions)
Contact (/contact)
Feedback
Terms of Use (/legal/terms)
Privacy Policy (/legal/privacy)

OpenReview (/about) is a long-term project to advance science through improved peer review, with legal nonprofit status through Code for Science & Society (https://codeforscience.org/). We gratefully acknowledge the support of the OpenReview Sponsors (/sponsors). © 2024 OpenReview
